# Host-Based Prognostic Biomarkers to Improve Risk Stratification and Outcome of Febrile Children in Low- and Middle-Income Countries

**DOI:** 10.3389/fped.2020.552083

**Published:** 2020-09-18

**Authors:** Núria Balanza, Clara Erice, Michelle Ngai, Rosauro Varo, Kevin C. Kain, Quique Bassat

**Affiliations:** ^1^ISGlobal, Hospital Clínic - Universitat de Barcelona, Barcelona, Spain; ^2^Sandra-Rotman Centre for Global Health, Toronto General Research Institute, University Health Network-Toronto General Hospital, Toronto, ON, Canada; ^3^Tropical Disease Unit, Division of Infectious Diseases, Department of Medicine, University of Toronto, Toronto, ON, Canada; ^4^Centro de Investigação em Saúde de Manhiça, Manhiça, Mozambique; ^5^ICREA, Barcelona, Spain; ^6^Pediatric Infectious Diseases Unit, Pediatrics Department, Hospital Sant Joan de Déu (University of Barcelona), Barcelona, Spain; ^7^Consorcio de Investigación Biomédica en Red de Epidemiología y Salud Pública (CIBERESP), Madrid, Spain

**Keywords:** febrile syndrome, severe infection, sepsis, risk stratification, prognostic host-biomarkers, angiopoietin-2 (Ang-2), soluble triggering receptor expressed on myeloid cells 1 (sTREM-1)

## Abstract

Fever is one of the leading causes for pediatric medical consultation and the most common symptom at clinical presentation in low- and middle-income countries (LMICs). Most febrile episodes are due to self-limited infections, but a small proportion of children will develop life-threatening infections. The early recognition of children who have or are progressing to a critical illness among all febrile cases is challenging, and there are currently no objective and quantitative tools to do so. This results in increased morbidity and mortality among children with impending life-threatening infections, whilst contributing to the unnecessary prescription of antibiotics, overwhelming health care facilities, and harm to patients receiving avoidable antimicrobial treatment. Specific fever origin is difficult to ascertain and co-infections in LMICs are common. However, many severe infections share common pathways of host injury irrespective of etiology, including immune and endothelial activation that contribute to the pathobiology of sepsis (i.e., pathogen “agnostic” mechanisms of disease). Importantly, mediators of these pathways are independent markers of disease severity and outcome. We propose that measuring circulating levels of these factors can provide quantitative and objective evidence to: enable early recognition of severe infection; guide patient triage and management; enhance post-discharge risk stratification and follow up; and mitigate potential gender bias in clinical decisions. Here, we review the clinical and biological evidence supporting the clinical utility of host immune and endothelial activation biomarkers as components of novel rapid triage tests, and discuss the challenges and needs for developing and implementing such tools.

## Introduction

In low- and middle-income countries (LMICs), it is common to have multiple infections throughout childhood that often manifest as febrile syndromes. It is estimated that a child under five years in a LMIC may experience around six episodes of fever per year (1). Consequently, fever is one of the leading causes for medical consultation and the most common pediatric symptom at clinical presentation (2). The vast majority of febrile illnesses are uncomplicated and self-limited. However, a small proportion of these may become severe and progress to sepsis, which is defined as life-threatening organ dysfunction due to a dysregulated host response to infection and considered to be agnostic to etiology (3, 4). While severe disease/sepsis needs urgent referral/admission and adequate treatment, febrile children without critical illness can be treated conservatively and often without antimicrobials once malaria has been ruled out (5, 6). The early recognition of children who have or are progressing to sepsis is challenging, because the initial presentation of a severe infection may be subtle and non-specific (2). Delays in the recognition and treatment of severe infections/sepsis increase child mortality and neurocognitive impairment in survivors (7, 8). Moreover, unnecessary admissions, referrals, and treatment of self-limited infections results in misallocation of health resources, overloading of health care facilities, contribution to development of antimicrobial resistance, and adverse events due to nosocomial infections and exposure to unnecessary investigations and treatments.

The underlying infectious etiologies of pediatric febrile syndromes are highly diverse and differ according to community (e.g., geographical location, season, or disease control measures implemented) and individual factors (e.g., age, immunity, or concomitant conditions) (2). Despite progress in recent years, the epidemiology of fever in LMICs remains poorly characterized (9, 10). The scarcity of available diagnostic tools and laboratory services in LMICs makes it challenging to establish whether a fever is viral, bacterial or parasitic in origin. Moreover, mixed or multiple infections, and high rates of asymptomatic pathogen carriers, are frequent in these settings (5, 11). The innovative use of minimally-invasive post-mortem methods, to investigate cause of death, also supports the co-existence of multiple infections and a progressive abandonment of the dogma “one pathogen—one disease—one cause of death” (12).

International guidelines for the management of childhood illnesses (i.e., Integrated Management of Childhood Illness [IMCI], integrated Community Case Management [iCCM]), which have been shown to improve child survival, give simplified algorithms to assist health care providers in fever management (13, 14). However, these are designed in such a broad way that may result in over-diagnosis, over-treatment, and over-referral (2). Pediatric disease severity scores (e.g., Lambaréné Organ Dysfunction Score [LODS] (15), Signs of Inflammation in Child that Kill [SICK] (16)) have also been developed, but their performance is highly dependent on the capacity of health care providers to identify danger signs and the availability of clinical and laboratory data. In parallel, current rapid diagnostic tests (RDTs) are largely pathogen-based (e.g., dengue and malaria RDTs) and, while they can identify the presence or absence of infection, they do not have the ability to differentiate between an uncomplicated infection vs. a life-threatening one. Previous studies have reported sub-optimal management of severe febrile syndromes in children, including poor triage, referral, and admission practices, in different levels of healthcare (17–20).

Additionally, once a child is admitted in a health facility, it is still difficult to clearly determine disease prognosis and thus make adequate decisions in regards to discharge. In fact, death in the first weeks after discharge remains an important and poorly acknowledged contributor to child mortality in LMICs, and can be as common or even more frequent than in-hospital mortality (21). Although triaging algorithms that identify children at increased risk of post-discharge mortality exist (22, 23), it is necessary to enhance how discharge decisions are made and identify those children that could benefit most from a follow-up visit.

Consequently, there is a compelling need to improve risk stratification tools for febrile children, for both initial management and discharge decisions. Accordingly, the World Health Organization (WHO) has highlighted the importance of developing new point-of-care (POC) tests to identify febrile patients at risk of progressing to severe disease/sepsis (2). This perspective article aims to highlight the potential of using host-biomarkers of disease severity and prognosis as objective measurements to guide critical clinical decisions. The measurement of these biomarkers using POC tests could complement clinical scores in community settings, as well as add value to them in formal health care settings.

### Pathogen “Agnostic” Mechanisms of Severe Infection

Host response has an important role in determining severity and outcome of infections. Moreover, it has become well-recognized that many different types of pathogens share common host response pathways of injury (i.e., pathogen “agnostic” mechanisms of disease) (24–26). Immune and endothelial activation lay upstream of endothelial destabilization, microvascular leak, multi-organ dysfunction, and death and, therefore, these pathways have been implicated in the pathogenesis of sepsis (27–29). A healthy endothelium must be adaptive in order to interact with its changing environment, alternating between activated and quiescent states by responding to host-derived cues. During an infection, the immune response triggers endothelium activation, meaning it becomes permeable and pro-inflammatory, in order to accommodate pathogen sequestration and elimination as well as vascular remodeling and healing. However, a dysregulated host response, in either step of this cascade of events, can result in endothelial dysfunction and tissue injury (25).

Evidence suggests that mediators of immune and endothelial activation are independent and quantitative markers of disease severity and prognosis in sepsis due to multiple causes, including: malaria, bacterial infections, dengue, or Ebola (25, 26, 28, 30–34). Therefore, measuring circulating levels of mediators of these pathways could be used for disease severity and prognostic determination to guide clinical decisions. Additionally, they represent an alternative to using acute phase proteins procalcitonin (PCT) and C-reactive protein (CRP). The performance of PCT and CRP as disease severity biomarkers has been extensively studied in high-income countries (HIC), but less is known from LMIC populations (35). In children living in LMICs, PCT and CRP concentrations have been reported to be influenced by prevalent conditions like malnutrition, coexisting malaria infection, and HIV-related immunosuppression (36–38).

### Mediators of Immune and Endothelial Activation as Prognostic and Severity Markers: Overview of Clinical Studies

**Figure 1:**
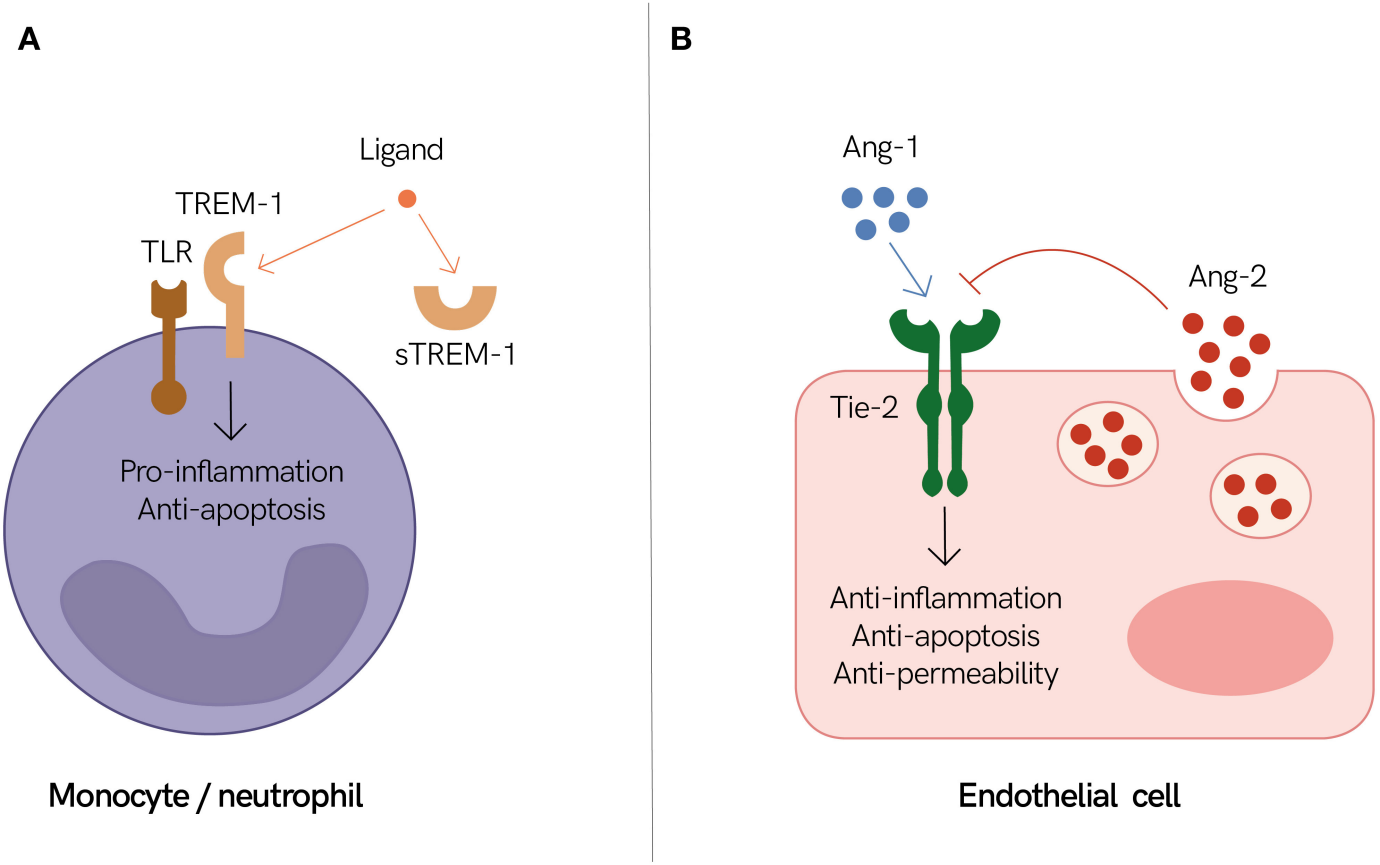
Endothelial and immune mediators as prognostic biomarkers of severe infections. **(A)** Triggering receptor expressed on myeloid cells 1 (TREM-1) together with Toll-like receptors (TLR) promote inflammation during an active infection. It is speculated that high levels of circulating soluble TREM-1 (sTREM-1) inhibit TREM-1 signaling by sequestrating its ligand. Therefore, individuals with high levels of circulating sTREM-1 may have a dysregulated immune response. **(B)** The endothelium shifts between states of quiescence (i.e., stable endothelium) and activation (i.e., permeable endothelium) to adapt and accommodate pathogen sequestration and elimination, as well as vascular remodeling and healing. The angiopoietin and tyrosine-protein kinase receptor (Ang/Tie) axis plays an important role in regulating these events. Angiopoietin-1 (Ang-1) activates Tie-2, which promotes endothelium stabilization, while angiopoietin-2 (Ang-2) inhibits these events, thereby promoting endothelium activation. Individuals with high levels of circulating Ang-2 may suffer from a dysregulated host response to infection resulting in excessive endothelium activation and vascular leak. **(A,B)** Both sTREM-1 and Ang-2 are associated with poor outcomes in different infectious diseases and thus are good candidates to help identify individuals with impending life-threatening infections.

A large body of literature exists for the potential use of immune and endothelial host-biomarkers as disease severity and prognostic tools. Here, we highlight and provide an overview of some of these biomarkers and concentrate on two specific promising examples (Figure 1).

Tumor necrosis factor (TNF) is the classic pro-inflammatory cytokine. Not surprisingly, elevated levels of TNF are observed in individuals with severe disease (i.e., severe malaria, bacteremia, and dengue) in comparison to individuals with uncomplicated disease (39–52). Increased TNF levels are also associated with neurological deficits and longer coma duration in children with cerebral malaria (53). Similarly, several studies have reported that individuals with severe disease and/or non-survivors have higher levels of soluble TNF receptor 1 (sTNFR1) (30, 47, 48, 54–61). Likewise, interleukins (ILs) are key components of the immune system and circulating concentrations of IL-6, IL-8, and IL-10 have been extensively studied in disease severity and progression. Among them, IL-6 is perhaps the best characterized. Several lines of evidence indicate that high IL-6 levels are correlated with bacterial sepsis, severe malaria, severe dengue, and increased risk of dengue mortality (39, 41, 44, 46, 48, 50, 62–68). A study in febrile adults reported IL-6 to be a good predictor analyte between non-survivors and survivors (54). Interferon γ-induced protein 10 (IP-10) is an interesting pro-inflammatory chemokine, but studies of its association with disease severity have generated contradictory results. A few studies have found reduced IP-10 levels in severe dengue cases, but other observed the contrary (69–72). In the context of malaria, studies have reported an association between high IP-10 and mortality (73–75), while in febrile adults low levels were associated with death (54). Chitinase-3-like 1 (CHI3L1) has also been identified as a severity marker in different causes of sepsis, with increased levels associated with severe disease, complications and mortality (54, 76, 77).

Molecules indicative of endothelial activation and dysfunction include mediators of endothelial cell function, components of the coagulation pathway, soluble cell surface adhesion molecules, and regulators of vascular tone and permeability (25). Among them, a growing body of evidence demonstrates that elevated levels of soluble intercellular adhesion molecule-1 (sICAM-1) are associated with disease severity and prognosis in malaria, dengue, and other causes of sepsis (72–74, 78–80). Soluble Fms-like tyrosine kinase 1 (sFlt-1) is another interesting marker of endothelial activation, which binds to vascular endothelial growth factor (VEGF). sFlt-1 has been reported to be good at discriminating between survivors and non-survivors in pediatric severe malaria (73, 74) and at predicting 28-day mortality in febrile adults (54). Moreover, other studies have shown that sFlt-1 correlates with severity in patients with bacterial sepsis (81).

### sTREM-1 as a Promising Biomarker of a Dysregulated Immune Response

Triggering receptor expressed on myeloid cells 1 (TREM-1) is expressed on neutrophils and monocytes. Activation of TREM-1 signaling culminates in an enhanced pro-inflammatory response and induction of anti-apoptotic pathways. Soluble TREM-1 (sTREM-1) has the same extracellular region as TREM-1 but lacks its transmembrane region. Thus, sTREM-1 can combine with the same ligands, and it is thought to negatively regulate TREM-1 signaling pathways (Figure 1A) (82).

Several clinical studies show that increased sTREM-1 levels are correlated with disease severity, longer clinical recovery times, multi-organ dysfunction, and mortality in both children and adults (54, 61, 73, 74, 83–86). It is speculated that these adverse outcomes are reflective of an imbalance in TREM-1 signaling, resulting in excess immune effector cells death and immunosuppression (54). ROC curve analysis of a cohort study conducted in Cameroon showed that sTREM-1 had discriminatory power between children with severe malaria vs. uncomplicated malaria (AUC = 0.96 [95% CI: 0.92–0.99]) (85). This finding is complemented by a Tanzanian study in outpatient febrile adults, which found that sTREM-1 is a strong prognostic marker for 28-day mortality (AUC = 0.87 [95% CI: 0.81–0.92]) (54). Importantly, this same study showed that the mortality prognostic utility of sTREM-1 was superior to CRP and PCT (*p* ≤ 0.0001). Another study conducted in rural Thailand in hospitalized patients with suspected infection also reported sTREM-1 to have strong prognostic 28-day mortality (AUC = 0.81 [95% CI: 0.77–0.85]) (61). Moreover, in these two studies, models combining sTREM-1 levels and validated clinical scoring systems (e.g., quick Sequential Organ Failure Assessment [qSOFA] and systemic Inflammatory Response Syndrome [SIRS]) had better prognostic accuracy than the clinical scoring systems alone (54, 61).

### The Ang/Tie Axis as an Example of Endothelial Integrity Modulator

Microvascular function and permeability are regulated by several pathways, including the angiopoietin and tyrosine-protein kinase receptor (Ang/Tie) axis. Angiopoietin-1 (Ang-1) and angiopoietin-2 (Ang-2) are ligands of the Tie-2 receptor, which is present on endothelial cells. Ang-1 is constitutively produced and secreted by pericytes and smooth muscle cells. Upon Ang-1 interaction with Tie-2, stabilization and maturation of blood vessels is triggered. Ang-2 is constitutively expressed at low levels and is co-localized with von Willebrand factor (vWF) within the Weibel Palade bodies (WPB) of endothelial cells. During an inflammatory response, both the expression of Ang-2 and its release from the WPB are increased, leading to an increased Ang-2:Ang-1 ratio. Ang-2 antagonizes Ang-1 downstream signaling by binding to Tie-2, resulting in endothelial destabilization, microvascular leakage, and organ dysfunction, which are common features of sepsis due to multiple causes (Figure 1B) (26).

Several studies have linked a dysregulated Ang/Tie axis to disease severity in malaria. Low levels of Ang-1, high levels of Ang-2, high Ang-2:Ang-1 ratios, as well as high soluble Tie-2 (sTie-2) levels are consistently reported in severe malaria in comparison to uncomplicated malaria (73, 78, 87–91). Elevated levels of Ang-2 and sTie-2 are associated with mortality, and Ang-2 has also been linked to multi-organ dysfunction, respiratory distress, impaired consciousness, and acute kidney injury among other complications (73, 74, 92–95). ROC curve analyses have demonstrated that Ang-1, Ang-2, and the ratio of Ang-2:Ang-1 have strong predictive power for disease severity and adverse outcomes, and some studies indicate that these markers are superior to lactate or parasitemia (73, 89, 91, 92). Interestingly, Ang-2 levels at presentation have also been reported to be good at predicting post-discharge mortality (AUC = 0.82 [95% CI: 0.71–0.93]) (74). Moreover, Ang-2 levels in combination with clinical predictors, have been shown to improve prognostic accuracy above that of clinical predictors alone (e.g., LODS) (74, 92). Levels of Ang-1, Ang-2, and sTie-2 have also been examined in other causes of sepsis. Results are mostly comparable to the malaria studies, as elevated Ang-2 and reduced Ang-1 levels have been associated with disease severity, intensive care unit (ICU) admission, shock, organ dysfunction, and death (54, 61, 79, 96–99).

## Challenges Ahead and the Need to Develop a Rapid Triage Tool

In the last decades, the number of published studies on biomarkers has increased exponentially. Recent scientific advances in high throughput “omics” technologies have driven the biomarker discovery and development field forward. Despite progress in the field and investment from major stakeholders, the number of biomarkers approved for any type of clinical use is limited (100, 101). Currently, the aforementioned biomarkers of immunological and endothelial origin hold promise, but they are not used in routine clinical practice in LMICs for risk-stratification of children.

For future implementation, further research is necessary. Additional scientific evidence is needed regarding which biomarkers are most useful and which cut-off levels should be used. The ideal prognostic biomarker for severe infection/sepsis should be: capable of identifying the subset of patients at high risk of adverse outcomes before this risk is clearly evident, correlated with disease severity over time, and useful regardless of disease microbiological etiology (25). Of the previously outlined biomarkers, sTREM-1 and Ang-2 are promising contenders to approximate this ideal. Besides, a combinational biomarker approach should also be considered, as it has been reported to enhance prognostic accuracy in some studies and it could be especially useful if biomarkers from different pathobiological pathways were measured (59, 73). Furthermore, rigorous and adequately powered randomized controlled trials will be essential to validate the clinical utility of adding biomarker measurements in guiding management decisions in both community-based and formal health care settings. Ideally, these studies should be carried out in different geographical areas in order to demonstrate reproducibility of findings and their added benefit to standard of care. If solid evidence of clinical benefit is provided, results could be used to adapt current international guidelines (i.e., iCCM, IMCI) (35).

Another crucial step is the development of rapid POC tests. This would allow measuring prognostic biomarkers at the community and peripheral level, where they could have a significant impact. These new rapid triage tests should fulfill the WHO ASSURED criteria which include simplicity, low cost, rapidness and suitability for field use (102). Recently, a lateral flow immunoassay has been reported to detect IL-6 in a quantitatively manner and similar devices need to be developed (103). Malaria RDTs are already widely implemented in Asia and Africa for fever management, where they are culturally accepted by communities (104–107). Therefore, it is expected that analogous devices would also be culturally acceptable. Another approach would be the integration of prognostic biomarker measurements into existing malaria RDTs for use in malaria endemic areas, rather than developing separate rapid triage tests, as proposed by McDonald et al. (24). In both scenarios a training phase for health care providers would be important to ensure their correct usage and interpretation of results.

Finally, cost-effectiveness studies are also necessary to ascertain affordability of using these specific biomarkers in different use case scenarios during routine clinical practice in LMICs. Currently, there are no cost-effectiveness analyses for the biomarkers we propose and therefore they need to be conducted. These will need to take into account the potential deaths, disability, adverse effects, and resource misallocation that could be averted. Importantly, in order to make this prognostic biomarker approach cost-effective, developing inexpensive rapid triage tests is essential.

## Broad Potential Impact

**Figure 2:**
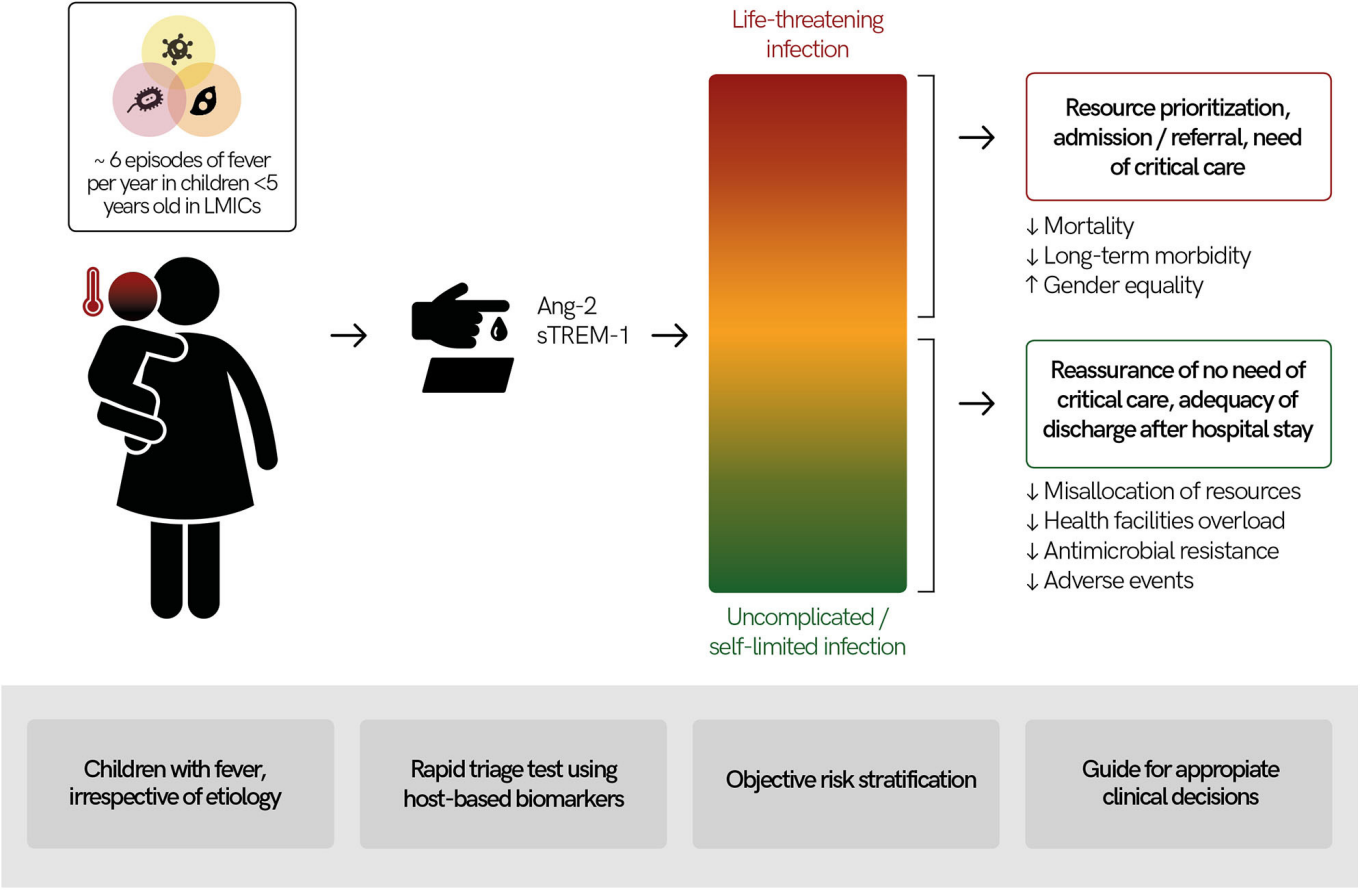
Usefulness of a rapid triage “traffic light” test using host-based biomarkers for febrile children in low- and middle-income countries (LMICs). Febrile illnesses are among the most common causes for children to seek medical services in LMICs and their etiologies are diverse. Measuring host-based prognostic biomarkers for disease severity (e.g., angiopoietin-2 [Ang-2], soluble triggering receptor expressed on myeloid cells 1 [sTREM-1]) in febrile children could lead to objective child risk-stratification. Results of a rapid triage test at point-of-care would provide actionable results for health workers, guiding appropriate clinical decision-making. This has the potential to lead to better health outcomes as well as economic savings.

If current barriers are overcome, rapid triage tools measuring host-biomarkers of immunological and endothelial origin have the potential to transform child fever management in LMICs. Objective risk stratification of febrile children could bring benefits for child health and optimize the use of limited health resources (Figure 2). Several studies have reported that the roll out of malaria RDTs has resulted in reduced antimalarial overuse but increased antibiotic prescription for febrile syndromes (108). Antimicrobial stewardship programs aim to reduce antimicrobial resistance by, among others, optimizing antibiotic use (109). Therefore, identification of individuals with life-threatening infections from those with self-resolving infections could help guide the safe reduction of antibiotic prescriptions.

The proposed approach could also reduce inequalities in fever management related to gender. Gender-based health disparities have been reported in LMICs, especially in south Asia, and are attributed to societal norms and family practices that favor sons (110, 111). These include differences in length of time before seeking care, frequency in consulting a health professional during illness, type of treatment given, or duration of hospital stay. This is a major driver of adverse health outcomes in girls and is hypothesized to contribute to the inequality observed in some countries' sex-stratified child mortality rates (112). A quantitative and objective triage tool indicative of severity could help mitigate gender biases when determining prioritization of clinical care, and referral/admission or discharge decisions.

## The Bigger Picture

Although the immediate benefit of host-biomarkers as prognostic tools is warranted for child risk stratification in LMICs, a similar triage tool for risk-stratification could also be useful in other scenarios, such as in pediatric health care settings in HIC and could also be applied to adult patient triage. In fact, some of the evidence we discuss comes from studies in HICs or from adults in LMICs. The current coronavirus disease (COVID-19) pandemic exemplifies the urgent need for accurate and rapid triage tools for early triage of severe disease, since most cases have self-limited courses but high-risk individuals need identification and prioritization of care and follow-up. HICs and LMICs are moving toward integrated approaches to care, and in both settings the ultimate aim is the same: better care whilst reducing costs.

## Conclusions

Mediators of endothelial and immunological pathways (e.g., sTREM-1 and Ang-2) have great potential as prognostic biomarkers for disease severity. Nevertheless, their clinical utility and cost-effectiveness needs further validation by prospective studies and randomized clinical trials. Their hypothetical implementation in LMICs via POC tests could provide actionable results for frontline health care workers facing the daily challenge of visiting children with fever. The proposed approach could be highly beneficial for child health, gender equality, appropriate use of antimicrobials, and optimization of limited health resources.

## Data Availability Statement

The original contributions presented in the study are included in the article/supplementary material, further inquiries can be directed to the corresponding author/s.

## Author Contributions

The manuscript was prepared with input from all authors. NB, CE, and MN contributed equally and share first co-authorship. KCK and QB share senior co-authorship. All authors read and approved the final manuscript.

## Conflict of Interest

KCK is a named inventor on a patent “Biomarkers for early determination of a critical or life-threatening response to illness and/or treatment response” held by the University Health Network. The remaining authors declare that the research was conducted in the absence of any commercial or financial relationships that could be construed as a potential conflict of interest.
